# Effects of mandibular advancement devices on upper airway dimensions in obstructive sleep apnea: responders versus non-responders

**DOI:** 10.1007/s00784-023-05186-w

**Published:** 2023-08-17

**Authors:** Xiaoxin Shi, Frank Lobbezoo, Hui Chen, Boudewijn R. A. M. Rosenmöller, Erwin Berkhout, Jan de Lange, Ghizlane Aarab

**Affiliations:** 1 Department of Orofacial Pain and Dysfunction, Academic Centre for Dentistry Amsterdam (ACTA), University of Amsterdam and Vrije Universiteit Amsterdam, Amsterdam, The Netherlands; 2 Department of Oral Radiology & Digital Dentistry, Academic Centre for Dentistry Amsterdam (ACTA), University of Amsterdam and Vrije Universiteit Amsterdam, Amsterdam, The Netherlands; 3 Department of Oral and Maxillofacial Surgery, Amsterdam University Medical Centers/Academic Centre for Dentistry Amsterdam (ACTA), University of Amsterdam, Amsterdam, The Netherlands; 4 Department of Orthodontics, School and Hospital of Stomatology, Cheeloo College of Medicine, Shandong University & Shandong Key Laboratory of Oral Tissue Regeneration & Shandong Engineering Laboratory for Dental Materials and Oral Tissue Regeneration, No. 44-1 Wenhua Road West, Jinan, 250012 Shandong China

**Keywords:** Obstructive sleep apnea, Mandibular advancement device, Cone beam computed tomography, Treatment response, Upper airway dimensions

## Abstract

**Study objectives:**

To compare the effects of mandibular advancement device (MAD) therapy on upper airway dimensions between responders and non-responders with mild to moderate obstructive sleep apnea (OSA).

**Methods:**

Thirty-one participants (21 men and 10 women) with a mean ± SD apnea-hypopnea index (AHI) of 16.6 ± 6.7 events/h, and aged 48.5 ± 13.9 years, were included in this study. Polysomnographic recordings and cone beam computed tomography (CBCT) scans in supine position were performed for every participant at baseline and at 3-month follow-up with their MAD in situ. Responders were defined as having ≥ 50% reduction in baseline AHI with a residual AHI < 10 events/h. The primary outcome variable was the minimal cross-sectional area of the upper airway (CSAmin).

**Results:**

No significant differences were found between responders (*n* = 15) and non-responders (*n* = 16) in age, gender distribution, body mass index, and neck circumference (*P* = 0.06–0.93), nor in AHI and CSAmin (*P* = 0.40 and 0.65, respectively) at baseline. The changes of the CSAmin with MAD in situ in the responder group were not significantly different compared to those in the non-responder group (*P* = 0.06).

**Conclusion:**

Within the limitations of this study, we conclude that the changes of the upper airway dimensions induced by MADs are not significantly different between responders and non-responders with mild to moderate OSA.

**Trial registration:**

ClinicalTrials.gov Identifier: NCT02724865. https://clinicaltrials.gov/ct2/show/NCT02724865

## Introduction

Obstructive sleep apnea (OSA) is a common sleep-related breathing disorder characterized by recurrent complete (i.e., apnea) and partial (i.e., hypopnea) obstructions of the upper airway, often resulting in oxygen desaturations and arousals from sleep [1, 2]. The diagnosis of OSA depends on either the presence of apnea-hypopnea index (AHI) ≥ 15 events/h, or an AHI ≥ 5 events/h accompanied by one or more of the symptoms such as excessive daytime sleepiness, fatigue, or impaired cognition [1, 2]. Most OSA patients have an impaired upper airway anatomy, which is also the key target of most existing treatments, such as continuous positive airway pressure (CPAP), upper airway surgery, weight loss, positional therapy, and mandibular advancement device (MAD) therapy [3, 4]. Compared to other treatment options, MADs are easy to use, non-invasive, less expensive, and have similar treatment effects as CPAP in mild to moderate cases [5, 6]. However, the efficacy of MAD therapy is variable and unpredictable, with approximately 50% non-responders [7–9] when using the recommended success criterion (i.e., ≥ 50% reduction in AHI with post-treatment AHI < 10 events/h) [10]. At this moment, the mechanism underlying different responses is not fully understood [11]. Therefore, there is an ongoing interest in the underlying mechanism of inter-individual variability in treatment responses. This knowledge is important for selecting the best candidates for MAD therapy.

The rationale behind the efficacy of MADs is that advancement of the mandible and tongue improves upper airway patency during sleep by enlarging the upper airway and by decreasing upper airway collapsibility [12–16]. However, the changes of the upper airway dimensions with the same mandibular advancement may differ between individuals due to many factors, such as the mandible morphology [17] and the soft tissue structures around the upper airway [18]. Some cephalometric studies suggested that the improvement of the upper airway dimensions was only observed in responders [13, 14, 19]. However, the 2-D images used in those studies could only provide limited information about the actual 3-D upper airway structures and may have projection errors [20]. Based on 3-D imaging techniques, such as magnetic resonance imaging (MRI) and cone beam computed tomography (CBCT), the differences observed in the effects of MADs on upper airway dimensions between responders and non-responders are not consistent. A study of Chan et al. [21] indicated that the enlargement of the upper airway dimensions with MAD in situ is present in responders only. By contrast, other studies suggested that there is no significant difference in the changes of upper airway dimensions between both groups [22–24]. The different results could be explained by the different imaging techniques (MRI vs. CBCT), imaging posture (supine vs. upright), and the upper airway variables used. Besides, some of these studies have compared both groups in a sub-group analysis, with shortcomings of either having potential confounding factors (e.g., different baseline OSA severity) and/or small sample size. Consequently, whether the different effects of MAD on upper airway dimensions is one of the underlying mechanisms explaining responder and non-responder status has not yet been fully determined. Therefore, more evidence is needed to better understand the difference in the effects of MADs on upper airway dimensions between both groups.

Since the primary working mechanism of MAD is to improve the upper airway dimensions, we hypothesized the following: (1) with MAD in situ, responders will show a larger improvement of the upper airway dimensions compared to non-responders; and (2) the improvement of the upper airway dimensions is positively associated with the improvement in the AHI. Therefore, the primary aim of this study was to compare the effects of MAD therapy on the upper airway dimensions between responders and non-responders with mild to moderate OSA based on CBCT images in the supine position. The secondary aim was to investigate the correlations between the changes in upper airway dimensions and the changes in AHI in the total group.

## Methods

### Overview

This study was part of a randomized controlled trial, in which individuals diagnosed with mild to moderate OSA (5 ≤ AHI < 30 events/h) were recruited to compare the efficacy of two types of MADs [25]. This study was approved by the Medical Research Ethics Committee of the Academic Medical Center Amsterdam (AMC), the Netherlands (#: NL44085.018.13). The study was registered at clinicaltrials.gov (ClinicalTrials.gov identifier: NCT02724865). Written informed consent was obtained from all participants.

### Participants

Eligible patients, diagnosed with OSA at one of four sleep centers in the Netherlands (Onze Lieve Vrouwe Gasthuis Ziekenhuis, Nederlands Slaap Instituut, Medisch Centrum Jan van Goyen, and AMC), were referred to the department of Oral and Maxillofacial Surgery of the AMC to participate in the present study. The inclusion criteria were as follows: (1) ≥18 years old; (2) ability to speak, read, and write Dutch; (3) ability to follow-up; (4) ability to use a computer with internet connection for online questionnaires; (5) diagnosis with symptomatic mild or moderate OSA (5 ≤ apnea-hypopnea index (AHI) < 30 events/h) with at least two OSA symptoms (e.g., snoring, fragmented sleep, witnessed apneas, and/or excessive daytime sleepiness [26]); and (6) expected to maintain current lifestyle (e.g., sports, medicine, diet). The exclusion criteria were as follows: (1) untreated periodontal problems, dental pain, and/or a lack of retention possibilities for an MAD; (2) medication usage that could influence respiration or sleep; (3) evidence of respiratory/sleep disorders other than OSA (e.g., central sleep apnea syndrome); (4) systematic disorders based on medical history and examination (e.g., rheumatoid arthritis); (5) severe temporomandibular disorders based on a functional examination of the masticatory system; (6) coexistence of non-respiratory sleep disorders (e.g., insomnia, periodic limb movement disorder, or narcolepsy); (7) known medical history of mental retardation, memory disorders, or psychiatric disorders; (8) reversible morphological upper airway abnormalities (e.g., enlarged tonsils, deviated nasal septum, and/or inferior nasal turbinate hypertrophy); (9) inability to provide informed consent; (10) simultaneous use of other modalities to treat OSA; and/or (11) previous treatment with an MAD.

### MADs

Two types of MAD were used in this study, namely, MAD-H (Herbst appliance; 4Dental labs, Amsterdam, the Netherlands) and MAD-S (SomnoDent appliance; SomnoDent Flex, SomnoMed, Sydney, Australia). Both types of MAD were titratable, two-piece, custom-made MADs, and were randomly allocated to the participants [25].

The detailed titration protocol was described previously [27]. In short, both types of MAD were set at 60% of the maximal mandibular advancement at baseline, and titrated backwards or forwards based on a weighted compromise between subjective improvement and side-effects during a 3-month follow-up. No adjustments were made when the patient reported a sufficient efficacy without side effects.

### Side effects and compliance

Self-reported side effects were recorded at each clinical visit, including the following: (1) sensitive teeth in the morning; (2) painful jaw muscles; (3) painful temporomandibular joints; and (4) changed occlusion in the morning. Information about the adherence and satisfaction level was collected by a telephone survey, which were expressed as follows: (1) percentage of hours of MAD use per total sleep time, (2) percentage of days of MAD use per week, and (3) the overall level of satisfaction with the MAD usage.

### Polysomnographic (PSG) recordings

This study consisted of two PSG recordings for each participant: one at baseline without MAD and one at 3-month follow-up with MAD in situ. A digital PSG system (Embla A10, Broomfield, CO, USA) was used and recorded electroencephalogram (EEG) (FP2-C4/C4-O2), electrooculogram (EOG), electrocardiogram (ECG), and submental and anterior tibial electromyogram (EMG). Nasal airflow was measured by a nasal pressure cannula, and blood oxygen saturation was measured by finger pulse oximetry. Straps containing piezoelectric transducers recorded thoracoabdominal motion, and a position sensor (Sleepsense, St Charles, IL, USA) attached to the midline of the abdominal wall was used to differentiate between supine, prone, right lateral, left lateral, and upright positions [28, 29].

PSG parameters, including sleep and respiratory variables, were analyzed following the recommendations of the American Academy of Sleep Medicine [1]. Responders were defined as having ≥ 50% reduction in baseline AHI with a residual AHI <10 events/h at the time of therapy evaluation. If this criterion was not met, patients were regarded as non-responders [10].

### CBCT scans

Two CBCT scans (NewTom 5G, QR systems, Italy) were performed for every participant at the department of Oral Radiology of the Academic Centre for Dentistry Amsterdam (ACTA): one without MAD at baseline and one with MAD in situ at 3-month follow-up in the same protrusion position as during the follow-up PSG recording. CBCT scans were performed while the patient was awake in the supine position with quiet breathing. The head of the patient was positioned with the Frankfort plane being perpendicular to the floor. After CBCT scanning, we further standardized the head position, during which the palatal plane (anterior nasal spine (ANS)-posterior nasal spine (PNS)) was adjusted to be parallel to the axial plane and the sagittal plane, and perpendicular to the coronal plane [30]. CBCT datasets were saved as Digital Imaging and Communications in Medicine (DICOM) files for further analysis.

### Craniofacial characteristics

**Fig. 1:**
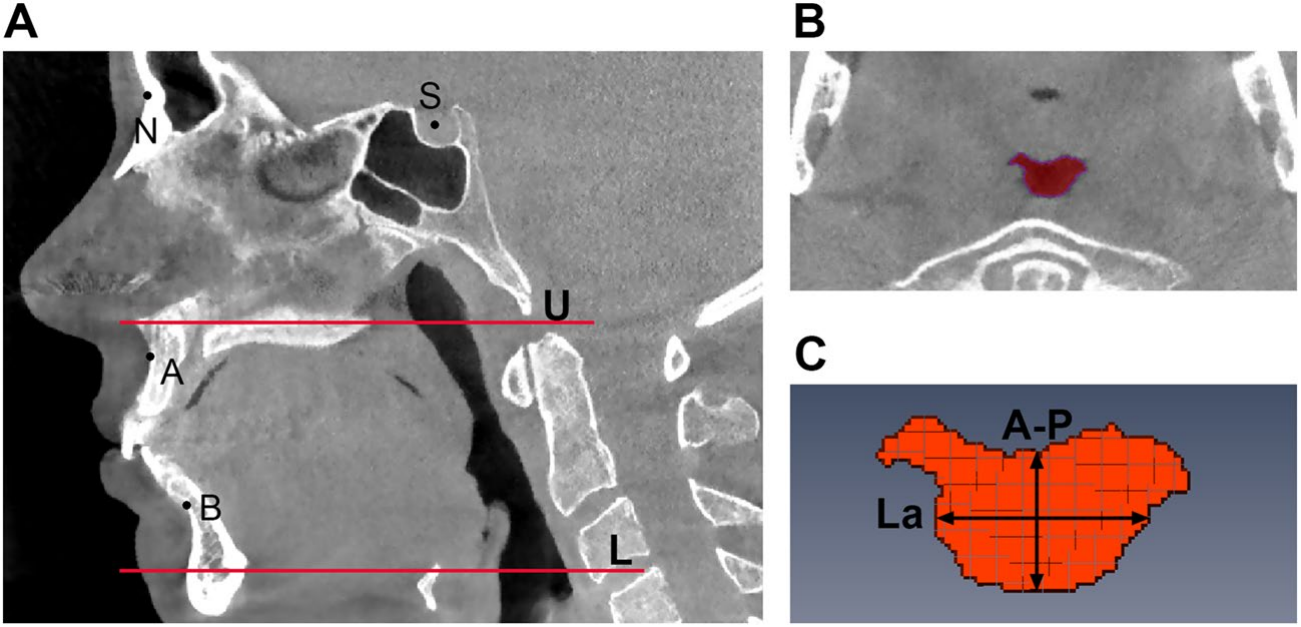
Craniofacial landmarks and measurements of the upper airway dimensions based on cone beam computed tomography (CBCT) imaging. **A** N = nasion, S = sella, A = subspinale, B = supramentale, U= the upper boundary of the upper airway (hard palate plane), L = the lower boundary of the upper airway (plane cross the base of the epiglottis) on the mid-sagittal plane. **B** The minimal cross-sectional area of the upper airway (CSAmin) on the axial plane. **C** The measurement of the anteroposterior dimension (A-P) and the lateral dimension (La) of the CSAmin

Measurements of the positions of the maxilla and mandible were performed using 3Diagnosys® software (v5.3.1, 3diemme, Cantu, Italy). The angle between sella, nasion, and subspinale (SNA angle), and the angle between sella, nasion, and supramentale (SNB angle) were used to represent the anteroposterior position of the maxilla and mandible relative to the cranial base, respectively. Landmarks are illustrated in Fig. 1A.

### Upper airway dimensions

The measurements of upper airway dimensions were performed using the Amira® software (v4.1, Visage Imaging Inc., Carlsbad, CA, USA). After setting the superior boundary of the upper airway (i.e., the palatal plane) and the inferior boundary (i.e., the horizontal plane across the base of the epiglottis) (Fig. 1A), the volume of the upper airway (V), the sequence number of each slice, and the cross-sectional area (CSA) of each slice were calculated automatically in the software [30]. Based on the CSA, the minimum CSA (CSAmin) could be identified and located (Fig. 1B), on which the anteroposterior dimension (A-P) and lateral dimension (La) of CSAmin could be measured (Fig. 1C). Based on the slice numbers of the upper airway, the length of upper airway (L) was calculated by multiplying the slice number with 0.3mm (the thickness of every slice). The investigator was blinded for the treatment response.

Since the CSAmin is suggested to be the most relevant anatomical characteristic related to the pathogenesis of OSA [31], the CSAmin was the primary outcome variable of the upper airway dimensions. The secondary upper airway variables included the lateral dimension (La) and anteroposterior dimension (A-P) of the CSAmin, upper airway volume (V), and upper airway length (L).

An experienced examiner who was blinded to the membership of the patients measured all the upper airway variables. Ten CBCT scans were randomly selected and re-measured after a 1-month interval of the original measurements, and the intra-rater reliability was assessed.

### Statistical analysis

Normality assumptions were verified with the Shapiro–Wilk test. Independent t test (for normally distributed variables), Mann-Whitney *U* test (for non-normally distributed variables), and Chi-squared test or Fisher’s exact test (for categorical variables) were used to compare the baseline demographic and craniofacial characteristics, as well as MAD type, mandibular protrusion, side effects, and adherence reports between the responder group and the non-responder group.

A two-way mixed, absolute agreement, single measures intraclass correlation coefficient (ICC) was used to determine the intra-rater reliability for the upper airway variables. Analyses of covariance (ANCOVA) were used to compare changes in sleep, respiratory, and upper airway variables between baseline and therapy evaluation between both groups. Significantly different baseline characteristics between responders and non-responders were controlled for as covariates [32]. Bonferroni-Holm method was used to correct the multiple comparisons of the secondary outcome variables of the upper airway [33]. Correlations between the changes in respiratory variables and the changes in upper airway dimensions were assessed using Pearson’s correlations (for normally distributed variables) or Spearman’s correlation (for non-normally distributed variables).

To test the robustness of the results of ANCOVA and correlation analyses, sensitivity analyses were performed by excluding the outliers of the primary variable (i.e., CSAmin) [34]. An outlier was defined as individual patients with the change of the primary variable (i.e., the change of CSAmin) more than 3 times the interquartile range (IQR) of the change above the 75^th^ percentile or below the 25^th^ percentile of all participants.

Statistical analysis was performed using the SPSS software (SPSS version 26, Chicago, IL, USA). A post hoc power analysis was conducted for the primary outcome variable (i.e., CSAmin) using software G*power (version 3.1.9, Franz Faul, Universität Kiel, Germany).

## Results

### Recruitment

Of the 49 patients who were initially recruited, 18 patients were excluded for reasons of lost contact (*n *= 5), quit study due to MAD complaints (*n *= 4), refused the second PSG recordings (*n *= 2), and with incomplete dataset (*n *= 7). Finally, 31 patients were included in this study: 15 of them were classified as responders, and 16 were non-responders.

### Patient characteristics

The baseline demographic and craniofacial characteristics of the responder group and the non-responder group are shown in Table 1. There were no significant differences between responders (*n* = 15) and non-responders (*n* = 16) in gender distribution, body mass index (BMI), neck circumference, and OSA severity categories (*P* = 0.61–0.93). Besides, there were no significant differences in SNA angle and SNB angle between both groups (*P* = 0.50 and 0.34, respectively). The responder group (43.7 ± 11.1 years) tended to be significantly younger than the non-responder group (52.9 ± 15.1 years) (*t* = −1.93, *P* = 0.06). Therefore, age was considered a covariate in the ANCOVA.

**Table 1:** Baseline demographic and craniofacial characteristics of the responder group and the non-responder group

	Responders (*n* = 15)	Non-responders (*n* = 16)	Test statistics	*P*
Demographic characteristics				
Age (years)	43.7 ± 11.1	52.9 ± 15.1	−1.93 (t)	0.06
Gender (men vs. women)	11 vs. 4	10 vs. 6	FET	0.70
BMI (kg/m^2^)	25.0|26.0|29.4	24.0|27.4|30.7	−0.38 (Z)	0.71
Neck circumference (cm)	38.9 ± 4.0	39.1 ± 4.0	−0.09 (t)	0.93
OSA severity (mild vs. moderate)	6 vs. 9	5 vs. 11	0.26(χ^2^)	0.61
Craniofacial characteristics				
SNA (°)	78.3|81.5|84.7	79.6|80.8|84.8	−0.67 (Z)	0.50
SNB (°)	76.7 ± 4.3	78.1 ± 3.8	−0.96 (t)	0.34

Normally distributed data are shown as mean ± standard deviation (SD); non-normally distributed data are presented as 25th percentile|median|75th percentile; *t*, independent t test; *Z*, Mann-Whitney *U* test; *FET*, Fisher’s exact test; *χ*^2^, Chi-squared test; *BMI*, body mass index; *mild OSA severity*, 5 ≤ AHI < 15 events/h; *moderate OSA severity*, 15 ≤ AHI < 30 events/h; *SNA*, angle between sella, nasion, and subspinale; *SNB*, angle between sella, nasion, and supramentale

### MAD use

The MAD type, amount of mandibular advancement, side effects, and adherence reports of the responder group and the non-responder group are presented in Table 2. There was no significant difference in MAD type used between both groups *(χ*^2^ = 0.03*, P* =0.85). Besides, no significant difference was found between both groups in the amount of mandible advancement in both percentage (%) and actual amount (mm) of the maximum protrusion (*P* = 0.34 and 0.12, respectively). No significant difference was found between both groups in their side effects either (*P* = 0.33–0.65). Nine patients in the responder group and 8 patients in the non-responder group responded on the adherence questionnaire. No significant differences in adherence reports were found between both groups (*P* = 0.29–0.86).

**Table 2:** MAD type, amount of mandibular advancement, side effects, and adherence reports of the responder group and the non-responder group

	Responders (*n =* 15)	Non-responders (*n =* 16)	*t*/*Z*/*χ*^2^/FET	*P*
MAD type				
MAD-H vs. MAD-S	8 vs. 7	8 vs. 8	0.03 (*χ*^2^)	0.85
Mandibular advancement				
Advancement (%)	70.0|75.0|90.0	62.5|75.0|75.0	−0.96 (*Z*)	0.34
Advancement (mm)	9.2 ± 2.5	8.0 ± 1.6	1.60 (*t*)	0.12
Side effects				
Sensitive teeth in the morning	3	2	FET	0.65
Painful jaw muscles	1	3	FET	0.60
Painful temporomandibular joints	4	2	FET	0.39
Changed occlusion in the morning	1	4	FET	0.33
Adherence	(*n*=9)^a^	(*n*=8) ^a^		
Wearing hour (%)	100.0|100.0|100.0	100.0|100.0|100.0	−0.17 (Z)	0.86
Wearing day (%)	100.0|100.0|100.0	100.0|100.0|100.0	−1.06 (Z)	0.29
Level of satisfaction (%)	68.9 ± 25.1	76.0 ± 16.1	−0.69 (t)	0.50

Normally distributed data are shown as mean ± standard deviation (SD); non-normally distributed data are presented as 25th percentile|median|75th percentile; *t*, independent t test; *Z*, Mann-Whitney *U* test; *χ*^2^, Chi-squared test; *FET*, Fisher’s exact test; ^a^compliance data were available for 9 patients of responder group and 8 patients of non-responder group

*MAD*, mandibular advancement device; *MAD*-*H*, MAD of Herbst type; *MAD*-*S*, MAD of SomnoDent type

### Reliability of the upper airway assessment

The intra-rater reliability for the upper airway assessment was excellent, with ICC = 0.96 for the CSAmin and ICC = 0.90 to 0.94 for the secondary outcome variables [35].

### Sleep and respiratory variables

The sleep and respiratory variables at baseline and at 3-month follow-up with MAD in situ of the responder and non-responder groups are shown in Table 3. The baseline values of the sleep and respiratory variables were not significantly different between both groups (*P* = 0.10–0.97). By definition, the reductions of AHI and AHI-supine were significantly larger in responders compared to non-responders (*P* < 0.01 and < 0.01, respectively). However, the changes of AHI-non-supine, oxygen desaturation index (ODI), and sleep variables were not significantly different between both groups (*P* = 0.16–0.93).

**Table 3:** Sleep and respiratory variables at baseline and at 3-month follow-up of the responder group and the non-responder group

	Responders (*n*=15)		Non-responders (*n*=16)		Baseline comparisons (ANCOVA)		Therapy effects comparisons (ANCOVA)	
	Baseline	3-month	Baseline	3-month	*F*	*P*	*F*	*P*
Sleep variables ^a^								
Total sleep time (min)	398.3 ± 53.9	416.7 ± 47.8	408.1 ± 75.0	406.2 ± 78.8	0.97	0.33	1.38	0.25
Stage N1 (%)	7.4 ± 5.2	6.0 ± 4.5	10.3 ± 4.7	8.3 ± 6.7	1.92	0.18	0.29	0.60
Stage N2 (%)	54.2 ± 7.5	49.9 ± 11.7	49.7 ± 9.3	45.9 ± 11.3	2.98	0.10	0.05	0.83
Stage N3 (%)	17.3 ± 7.4	19.7 ± 8.6	19.4 ± 5.8	23.5 ± 8.5	1.34	0.26	0.23	0.63
Stage REM (%)	21.1 ± 4.5	24.3 ± 5.8	20.6 ± 6.0	22.3 ± 7.4	0.00	0.97	0.21	0.65
Supine position (%)	44.4 ± 37.4	37.1 ± 27.3	39.9 ± 27.0	42.3 ± 27.6	0.00	0.97	0.76	0.39
Arousal index(/h)	7.9 ± 5.3	11.5 ± 11.8	11.8 ± 16.0	14.2 ± 13.5	0.87	0.36	0.01	0.93
Respiratory variables								
AHI (/h)	15.9 ± 6.1	4.9 ± 2.6	17.3 ± 7.3	17.1 ± 6.8	0.72	0.40	15.38	<0.01*
AHI-supine (/h)	29.8 ± 16.1	9.9 ± 10.9	30.4 ± 15.4	34.3 ± 22.5	0.00	0.97	10.76	<0.01*
AHI-non-supine (/h)	8.3 ± 8.2	3.2 ± 2.7	11.0 ± 8.5	7.1 ± 4.0	0.57	0.46	0.16	0.69
ODI (/h)	16.0 ± 8.9	8.0 ± 4.4	16.1 ± 11.3	15.0 ± 7.5	0.39	0.54	2.12	0.16

Data are shown as mean ± standard deviation (SD); *ANCOVA*, analysis of covariance using age as a covariate; ^a^two patients with incomplete sleep data were excluded from the analysis of sleep variables; *statistically significant

*REM*, rapid-eye-movement; *AHI*, apnea-hypopnea index; *AHI-supine*, AHI score in the supine position; *AHI*-*non*-*supin*e, AHI score in positions other than the supine position; *ODI*, oxygen desaturation index

### Upper airway variables

**Fig. 2:**
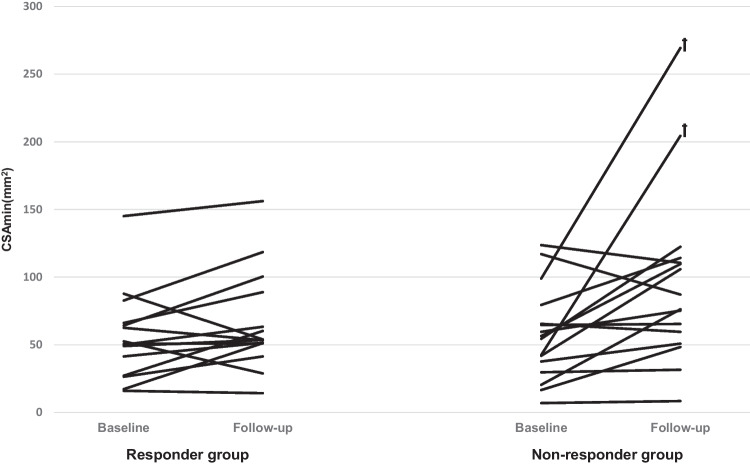
Individual values of the minimal cross-sectional area of the upper airway (CSAmin) at baseline and at follow-up in the responder group (*n =* 15) and the non-responder group (*n =* 16). No significant difference was observed in the changes in the CSAmin between both groups (*P* = 0.06). ꝉ: outliers

The variables of upper airway dimensions at baseline and at 3-month follow-up with MAD in situ in the responder group and the non-responder group are presented in Table 4. For the primary outcome variable, CSAmin, the enlargement of CSAmin with MAD in situ was not significantly different between both groups (*P* = 0.06). The individual values of the CSAmin at baseline and at follow-up in the responder and non-responder groups are illustrated in Fig. 2. For the secondary upper airway variables, there were no significant differences between both groups in the changes of A-P dimensions, La dimensions, upper airway volume, and upper airway length after Bonferroni-Holm correction (*P* = 0.04–0.18). Similar results were found in the sensitivity analyses of ANCOVA after excluding the two outliers in the non-responder group: there were no significant differences between both groups in the changes of the CSAmin (*P* = 0.20) and the secondary outcome variables (*P* = 0.11–0.54) either. The observed effect size f of the changes of CSAmin between both groups was 0.37 in the primary analysis (partial *η*^2^ = 0.12, ANCOVA) and 0.25 in the sensitivity analysis (partial *η*^2^ = 0.06, ANCOVA), which can be qualified as between medium and large.

**Table 4:** Variables of upper airway dimensions at baseline and at 3-month follow-up with MAD in situ of the responder and non-responder groups

	Responders (*n*=15)		Non-responders (*n*=16)		Baseline comparisons (ANCOVA)		Therapy effects comparisons (ANCOVA)	
	Baseline	3-month	Baseline	3-month	*F*	*P*	*F*	*P*
Primary outcome								
CSAmin (mm^2^)	55.9 ± 32.8	65.8 ± 36.3	57.2 ± 34.1	96.2 ± 64.6	0.22	0.65	3.80	0.06
Secondary outcomes								
A-P (mm)	4.8 ± 1.8	4.9 ± 1.5	4.3 ± 1.9	5.4 ± 2.6	1.32	0.26	1.89	0.18
La (mm)	12.1 ± 3.6	14.3 ± 4.3	12.3 ± 5.6	17.1 ± 6.0	0.02	0.89	3.61	0.07
V (cm^3^)	9.5 ± 2.9	10.0 ± 3.6	12.6 ± 5.1	14.9 ± 6.0	1.92	0.18	2.60	0.12
L (mm)	65.3 ± 9.5	62.4 ± 9.5	67.5 ± 8.1	67.4 ± 8.7	0.26	0.61	4.90	0.04

Data are shown as mean ± standard deviation (SD); *ANCOVA*, analysis of covariance using age as a covariate; *CSAmin*, minimum cross-sectional area of the upper airway; *A-P*, anteroposterior dimension of the CSAmin; *La*, lateral dimension of the CSAmin; *V*, volume of the upper airway; *L*, length of the upper airway

### Correlations

The correlations between the changes in upper airway dimensions and the changes in the respiratory variables of the total group are shown in Table 5. There were no significant correlations between the changes in CSAmin and the changes in AHI (*P* = 0.48), AHI-supine (*P* = 0.10), and AHI-non-supine (*P* = 0.59). Besides, no significant correlations were found between the changes in the secondary upper airway variables and the changes in the respiratory variables either (all *P* > 0.05). Similar results were found in the sensitivity analyses of correlation analyses after excluding the two outliers in the non-responder group: no significant correlations were found between the changes in upper airway dimensions and the changes in the respiratory variables (all *P* > 0.05).

**Table 5:** Correlations between the changes in upper airway dimensions and the changes in the respiratory variables of all participants with OSA (*n *= 31)

	ΔAHI		ΔAHI-supine		ΔAHI-non-supine	
	*r*	*P*	*r*	*P*	*r*	*P*
ΔCSAmin (mm^2^)	0.13	0.48	0.30	0.10	−0.10	0.59
ΔA-P (mm)	0.05	0.81	0.09	0.63	−0.21	0.27
ΔLa (mm)	0.18	0.32	0.27	0.15	0.07	0.73
Δ*V* (cm^3^)	0.26	0.15	0.25	0.18	−0.27	0.15
Δ*L* (mm)	0.30	0.10	0.13	0.48	0.02	0.90

*Δ*, the difference between the baseline value and follow-up value; r, correlation coefficient; *CSAmin*, minimum cross-sectional area of the upper airway; *A-P*, anteroposterior dimension of the CSAmin; *La*, lateral dimension of the CSAmin; *V,* volume of the upper airway; *L*, length of the upper airway; *AHI*, apnea-hypopnea index; *AHI*-*supine*, AHI score in the supine position; *AHI-non-supine*, AHI scores in positions other than the supine position

## Discussion

The objectives of this study were to compare the effects of MAD therapy on upper airway dimensions between responders and non-responders with mild to moderate OSA, and to determine the correlations between the changes in upper airway dimensions and the changes in AHI in the total group. The results indicated that the changes of the upper airway dimensions with MAD in situ were not significantly different between responders and non-responders in the supine position. Furthermore, the changes of the upper airway dimensions had no significant correlation with the changes in AHI.

### Comparisons of the therapy effects on upper airway dimensions

The changes of CSAmin induced by MAD were not significantly different between responders and non-responders in the present study. Besides the CSAmin, the secondary outcome variables, including the lateral dimensions and the anteroposterior dimensions of the CSAmin, upper airway volume, and upper airway length, were not significantly different between both groups either. Contrary to our results, Chan et al. [21] compared 47 responders and 22 non-responders based on MRI and found a significant increase in the CSAmin and other upper airway dimensions in responders only. However, in their study, the responders had significantly higher AHI and bigger SNB angle compared to the non-responders at baseline, which may bias the upper airway comparisons and may explain the different results as compared to our findings. Our results are similar to three other studies [22–24], which all suggested that there were no significant differences between responders and non-responders regarding the changes of upper airway dimensions based on 3-D images. However, the generalization of these three studies might be limited due to different aspects. In the study of Pahkala et al. [24], the measurements of the upper airway dimensions were based on upright CBCT images, which may represent different upper airway dimensions compared to those based on supine images [36]. The study of Ogawa et al. [23] compared the upper airway dimensions based on the mid-sagittal plane of a MRI image only, and in the study of Sutherland et al. [22], the comparisons were performed as a sub-group analysis with a small sample (12 responders vs. 6 non-responders). Therefore, our study confirmed the results of previous studies with larger sample size and different upper airway variables in the supine position. The outcomes suggest that the enlargement of the upper airway with MAD in situ in the supine position during waking state cannot explain treatment success of MAD treatment.

In the present study, the observed effect size f of the difference in the changes of CSAmin between both groups (0.37 and 0.25 in the primary and sensitivity analyses, respectively) was between medium and large (according to Cohen [37], an effect size *f* = 0.10 is small, *f* = 0.25 is medium, and *f* = 0.40 is large). With these effect sizes, if we enlarge our sample size to around 60–130, there might be a significant difference between both groups (with power 0.8 and 5% significance level). However, it is important to note is that there is a tendency that non-responders will have a greater enlargement of CSAmin compared to responders, which confirms the aforementioned speculation that the upper airway enlargement by itself cannot explain treatment success.

Our results suggest that the effects of MAD on upper airway dimensions are similar between responders and non-responders in the supine position, although the changes of AHI and AHI-supine differed significantly. The non-significant findings of comparisons of upper airway dimensions do not support our initial hypothesis. However, the multifactorial nature of the pathogenesis of OSA may possibly explain our findings. It has been recognized that besides anatomical factors, non-anatomical factors like impaired pharyngeal dilator muscle responsiveness, increased propensity for awakening during airway narrowing (low respiratory arousal threshold), and respiratory control instability (high loop gain) are also crucial determinants of OSA for many patients [4]. Therefore, we hypothesize that the mechanical enlargement of the upper airway dimensions is an important prerequisite for treatment success of MAD, but the finial treatment outcome may be mediated by non-anatomical factors. Limited by technique, the non-anatomical factors could not be investigated for responders and non-responders in the present study. However, previous studies have suggested that compared to non-responders, responders had a less collapsible pharynx and a more stable respiratory control system (i.e., lower loop gain) [38, 39], which is in line with our hypothesis. Future studies are warranted to test this hypothesis.

### Correlations

No significant correlations were found between the changes in upper airway dimensions and the changes in AHI. Furthermore, as the upper airway images were performed in the supine position, the correlations between the changes in upper airway dimensions and the changes in AHI-supine were also investigated, and no significant correlations were found either. Our results are consistent with other studies [14, 22, 40, 41], in which no significant linear correlations were found between the changes in AHI and the changes in upper airway dimensions. In contrast, a study of Camañes-Gonzalvo et al. [16] has found a significant correlation between the increase of the upper airway volume and the decrease of AHI. However, the correlation was only significant in the severe OSA group, and not in the mild to moderate groups [16], which corresponds with the findings in the present study. Since OSA pathogenesis is explained by both anatomical and non-anatomical factors [4], it is to be expected that the relationship between the changes in respiratory parameters and the changes in the upper airway dimensions is not linear. The absence of a significant correlation is also in line with our non-significant finding in the comparisons of upper airway changes between both groups, which suggests that the prediction of treatment response of MAD using upper airway changes based on awake CBCT is of limited value.

### Demographic characteristics

Many studies have hypothesized that some demographic and craniofacial characteristics could be helpful in predicting the treatment success of MAD therapy. In the present study, responders were younger than non-responders, which is consistent with previous studies [42–45]. According to a recent systematic review and meta-analysis study [10], other characteristics that may associate with MAD treatment success are lower BMI, smaller neck circumference, a shorter airway length, and a smaller baseline CSAmin. However, the results of this study did not confirm these findings, which may be related to the size and/or heterogeneity of our sample in our study. In general, no single characteristic may reliably predict a favorable MAD treatment outcome [46], which calls for more research in the recognition of non-responders by combining demographic characteristics, anatomical factors, and non-anatomical factors.

### Vertical opening

The MADs used in the present study are customized adjustable devices which allow some degree of mouth opening during sleep [25]. When using similar customized adjustable devices (allowing mouth opening) and the same success criterion, the treatment success rate of the present study (48%) is consistent with previous studies reporting a success rate of approximately 50% [7, 8, 38]. However, studies have shown that increased mouth opening during sleep may compromise the beneficial effects of MAD therapy on upper airway dimensions and the treatment efficacy [47, 48]. It has been suggested that using elastic bands restricting the mouth opening during sleep may help improve the treatment efficacy of these MADs [49, 50]. In the pilot study of Norrhem et al. [49], MADs with elastic bands markedly reduced the supine AHI in 2 severe OSA patients, however, there was no significant difference in the overall AHI reduction between MADs with or without elastic bands. In the study of Milano et al. [50], the treatment efficacy was significantly higher in the MADs with elastic bands than the MADs without elastic bands in positional OSA patients, which may not represent the general OSA population. Therefore, well-designed RCTs are needed to investigate whether restricting the mouth opening increases the efficacy of the MADs that allow mouth opening.

### Study limitations

The present study has several limitations. First, the CBCT scans were performed in awake state. Therefore, the upper airway morphology may not be the same as that in sleep state. However, acquiring a radiologic image of the upper airway during sleep is clinically challenging. Besides, as the baseline and follow-up CBCT images in our study were both taken in the supine position, the comparisons of the changes of upper airway between responders and non-responders were not biased. Second, two types of MAD were used in this study. However, our previous study has suggested that there was no significant difference between these two types of MAD in affecting PSG parameters and upper airway dimensions [25]. Furthermore, in both groups, similar numbers of both MAD types were used. Therefore, we believe only limited bias was caused by including these two types of MAD in our study. In addition, as this study recruited patients in an RCT study with another primary aim [25], we did not perform an a priori power analysis to calculate the sample size. The effect size analyses of the primary outcome variable indicated that with a larger sample size (*n =* 60–130 based on the primary and sensitivity analyses), non-responders tend to have a greater enlargement of CSAmin compared to responders.

## Conclusion

Within the limitations of this study, we conclude that the changes of the upper airway dimensions induced by MADs are not significantly different between responders and non-responders with mild to moderate OSA. Furthermore, the changes of the upper airway dimensions have no significant correlations with the changes in the apnea-hypopnea index.

## Data Availability

The datasets generated and/or analyzed during the current study are available from the corresponding author upon reasonable request.
